# Intelligence

**Published:** 1833-08

**Authors:** 


### INTELLIGENCE.

*Malignant Cholera.* Several cases of cholera, with all the characters of the formidable epidemic of last year, have recently occurred in London. As yet, however, it does not appear that the disease has any strong disposition to spread. A few cases have happened at Limehouse, where diarrhœa and dysentery are prevalent. Some cases have also ended fatally at Sunderland.

The streets of Bristol were a few days ago swarming with small black flies, in such immense numbers as in some degree to darken the atmosphere. The return of the cholera has, we regret to say, occurred at Bristol. The presence of myriads of black flies, and the prevalence of cholera, were occasionally noticed last year during the progress of the disease.

*The Concours at Paris.* The contest for the chair of *clinique interne* has at length closed, and M. ROSTAN has been appointed. There are many persons in this country who are extremely anxious that a *concours* should be established, as the best mode of determining the professional capabilities of candidates for medical offices in public institutions. If ever such a mode of examination should be adopted here, we trust it may be better managed than in Paris; where, during the election just terminated, as much confusion has been witnessed, and as much personal vituperation bandied about, as the most turbulent could desire. As a proof of the very great dissatisfaction that was felt, very strong protests and reclamations were presented to government against the issue of the last contest, when a new *concours* was opened at Paris for the chair of *pathologie externe* in the Faculty.

---

*College of Surgeons.* Mr. GUTHRIE has been elected president, and Messrs. WHITE and ANDREWS vice-presidents, for the ensuing year.

---

*Recognition of French Certificates at the Navy Board.* Sir WILLIAM BURNETT last week told a medical gentleman, who was on the eve of preparing for examination at the Navy Board, that, as there was great hindrance to the prosecution of anatomy at present in London, (from the want of subjects,) that he had better go to Paris to dissect; and that, if he did so, he (Sir William) would, on the return of the student to London, receive his French certificates at the Board.—*Lancet*, July 27.

---

### MONTHLY LIST OF MEDICAL BOOKS.

### METEOROLOGICAL REGISTER,

By Messrs. Harris and Co., Mathematical Instrument Makers, 50, High Holborn.

| | Moon. | Rain gauge. | Thermometer | | | Barometer. | | De Luc's Hygrometer | | Winds. | | Atmospheric Variation. | | |
|---|---|---|---|---|---|---|---|---|---|---|---|---|---|---|
| June | | | 9 a.m. | max. | min. | 9 a.m. | 10 p.m. | 9 a.m. | 10 p.m. | 9 a.m. | 10 p.m. | 9 a.m. | 2 p.m. | 10 p.m. |
| 20 | | .02 | 62 | 70 | 55 | 29.68 | 29.72 | 64 | 61 | NW | NNW | cloudy | fine | fine |
| 21 | | | 66 | 72 | 60 | .80 | .68 | 60 | 59 | NNW | WSW | fine | fine | cloudy |
| 22 | | | 65 | 69 | 58 | .74 | .60 | 59 | 58 | NNW | NW | | | rain |
| 23 | | | 62 | 67 | 46 | .36 | .38 | 59 | 62 | WSW | WNW | showery | rain | fine |
| 24 | ☽ | | 69 | 65 | 50 | .42 | .56 | 60 | 64 | W | W | cloudy | | |
| 25 | | .78 | 61 | 66 | 54 | .66 | .60 | 62 | 63 | WSW | N | fine | fine | |
| 26 | | | 62 | 68 | 56 | .52 | .42 | 63 | 66 | E | S | cloudy | | |
| 27 | | | 62 | 66 | 58 | .45 | .67 | 65 | 64 | WNW | SW | | | cloudy |
| 28 | | | 66 | 75 | 62 | .59 | .51 | 64 | 61 | SSW | WSW | fine | | fine |
| 29 | | | 68 | 69 | 55 | .55 | .62 | 59 | 57 | WSW | WSW | cloudy | | |
| 30 | | | 63 | 70 | 58 | .68 | .63 | 57 | 57 | WSW | SW | fine | | |
| July 1 | | | 64 | 65 | 49 | .58 | .60 | 58 | 61 | WSW | WNW | | | |
| 2 | ○ | .45 | 60 | 66 | 52 | .68 | .73 | 60 | 60 | WNW | WNW | overcast | fine | cloudy |
| 3 | | | 62 | 67 | 52 | 76 | .87 | 60 | 61 | WNW | NW | cloudy | | fine |
| 4 | | | 63 | 70 | 57 | .92 | .92 | 61 | 62 | W | SW | fine | | |
| 5 | | | 65 | 70 | 56 | .91 | .77 | 62 | 60 | WSW | SE | cloudy | | |
| 6 | | | 67 | 73 | 57 | .60 | .44 | 60 | 55 | SE | E | fine | | |
| 7 | | | 63 | 67 | 59 | .47 | .53 | 59 | 64 | SW | W | cloudy | rain | cloudy |
| 8 | | | 60 | 68 | 56 | .59 | .75 | 66 | 68 | WNW | ENE | rain | cloudy | fine |
| 9 | | .55 | 62 | 69 | 59 | .85 | .83 | 69 | 50 | NE | NNE | fine | fine | |
| 10 | ☾ | | 65 | 71 | 55 | .77 | .73 | 54 | 64 | NNE | NNE | | | |
| 11 | | | 64 | 71 | 57 | .67 | .65 | 64 | 65 | N | N | | | cloudy |
| 12 | | | 60 | 64 | 55 | .59 | .62 | 65 | 65 | SE | NE | cloudy | cloudy | |
| 13 | | | 58 | 63 | 54 | .67 | .76 | 65 | 65 | NNE | NNE | | | fine |
| 14 | | | 62 | 67 | 57 | .70 | .70 | 65 | 65 | SW | SW | | | |
| 15 | | | 64 | 70 | 58 | .76 | .87 | 65 | 63 | E | SE | | fine | |
| 16 | | | 66 | 72 | 61 | .96 | .95 | 62 | 61 | E | SSE | fine | | cloudy |
| 17 | ● | | 67 | 79 | 67 | .97 | .96 | 61 | 61 | SW | NE | | | |
| 18 | | | 71 | 78 | 64 | .90 | .80 | 61 | 59 | WNW | NW | | | overcast |
| 19 | | .45 | 69 | 73 | 62 | .63 | .51 | 59 | 61 | W | WSW | cloudy | | fine |

The quantity of Rain fallen in June, was 1 inch and 48-100ths.

